# Supplementary material for: Sanguinarine Induces Necroptosis of HCC by Targeting PKM2 Mediated Energy Metabolism
Source: Cancers (Basel). 2024 Jul 13;16(14):2533. doi: 10.3390/cancers16142533 (PMC11274805; doi:10.3390/cancers16142533)
Supplement: Supplementary file 1 [file cancers-16-02533-s001.zip › cancers-3051929-Figure S1.pdf]

Supplementary Materials

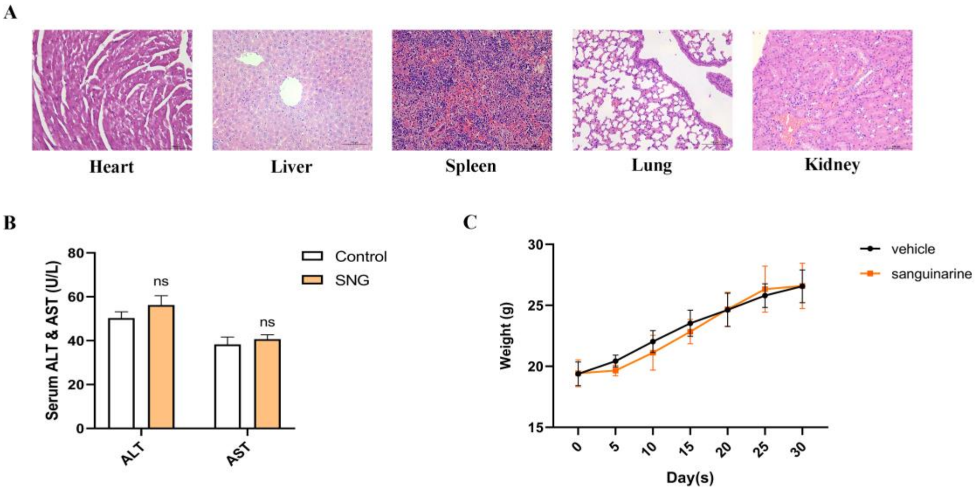

Figure S1 (A) H&E staining of heart, liver, spleen, lung and kidney from sanguinarine-treated mice. (B) Serum ALT and AST levels of vehicle group and sanguinarine (SNG) treated group. (C) Body weight of pretreatment, vehicle-treated and sanguinarine-treated mice.
