# Supplementary figures and images for: Sanguinarine Induces Necroptosis of HCC by Targeting PKM2 Mediated Energy Metabolism
Source: Cancers (Basel). 2024 Jul 13;16(14):2533. doi: 10.3390/cancers16142533 (PMC11274805; doi:10.3390/cancers16142533)

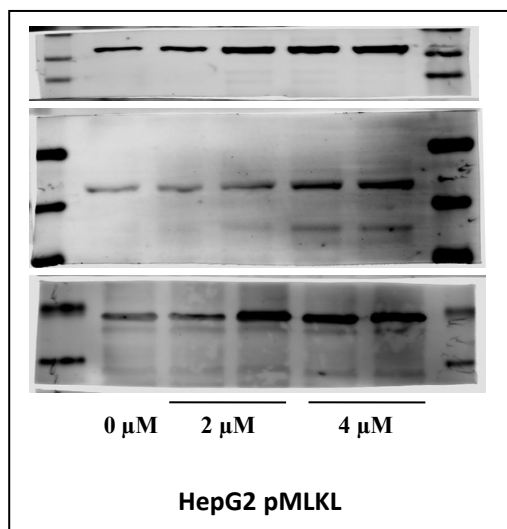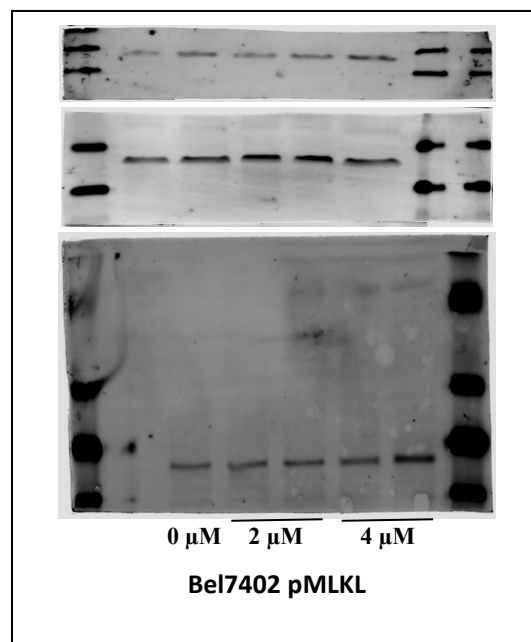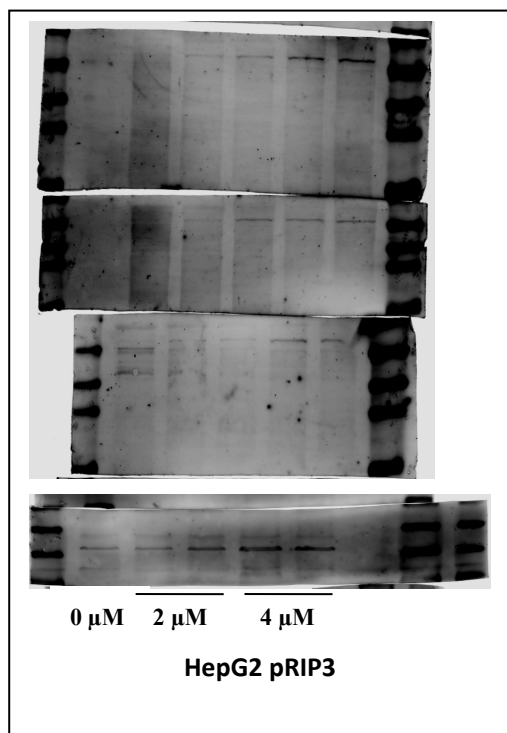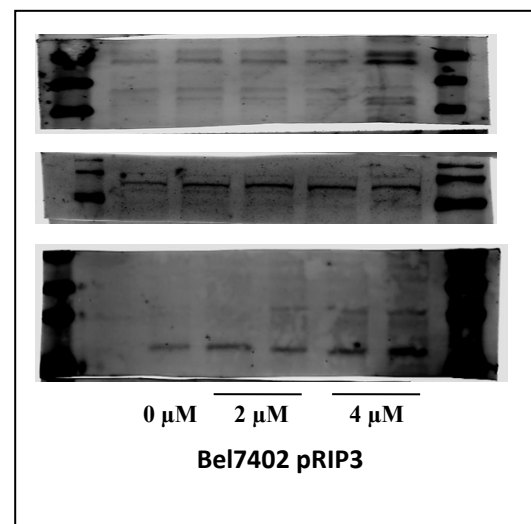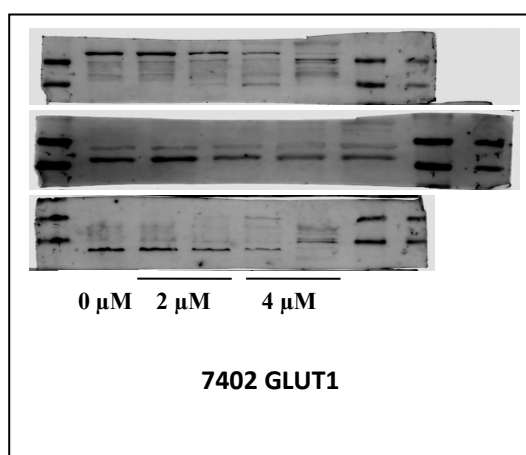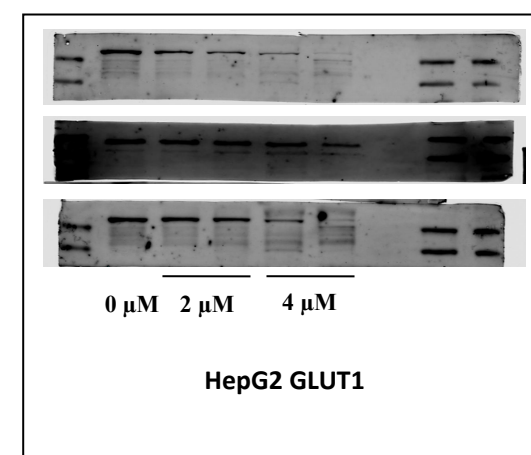

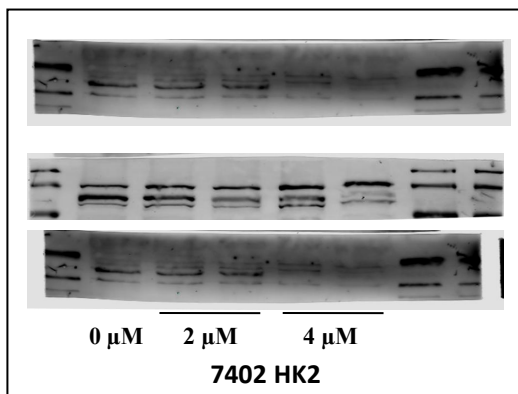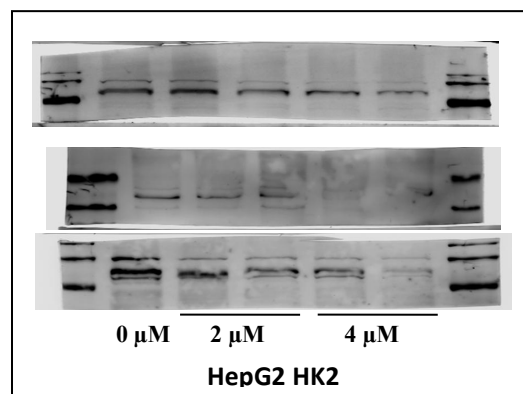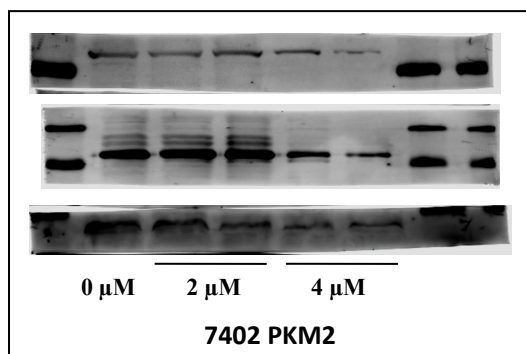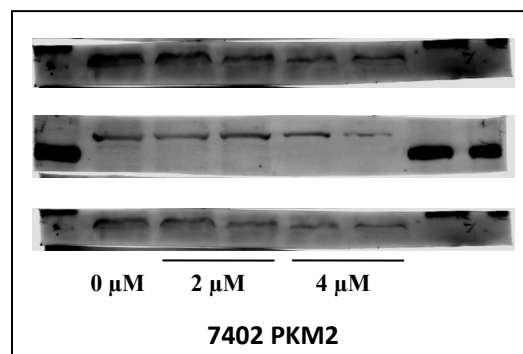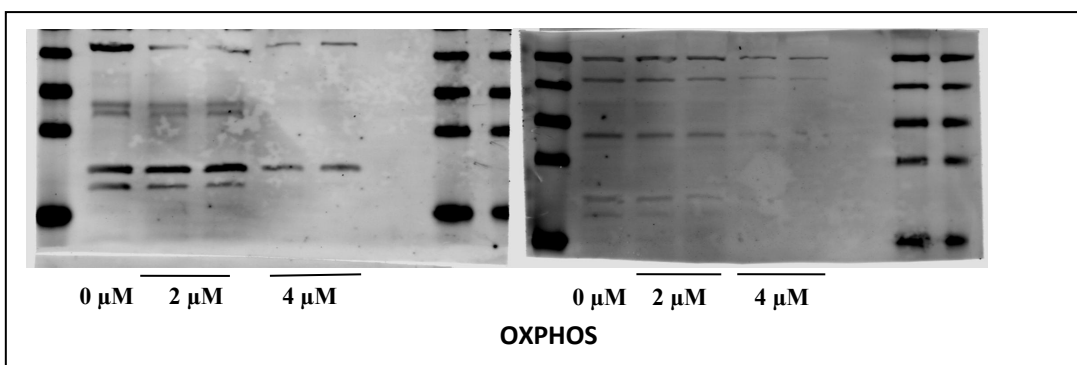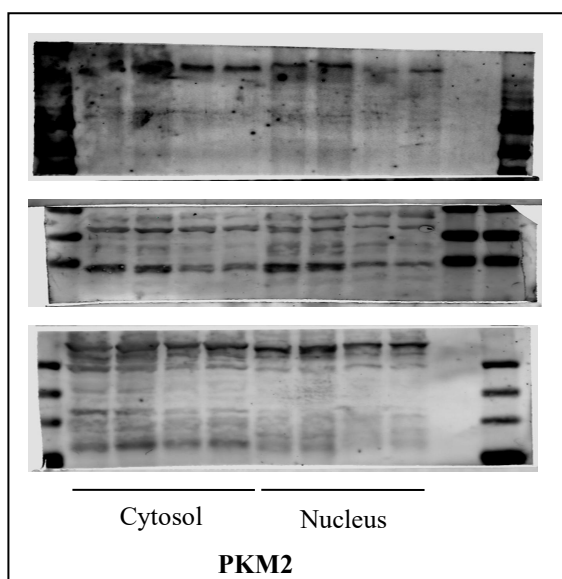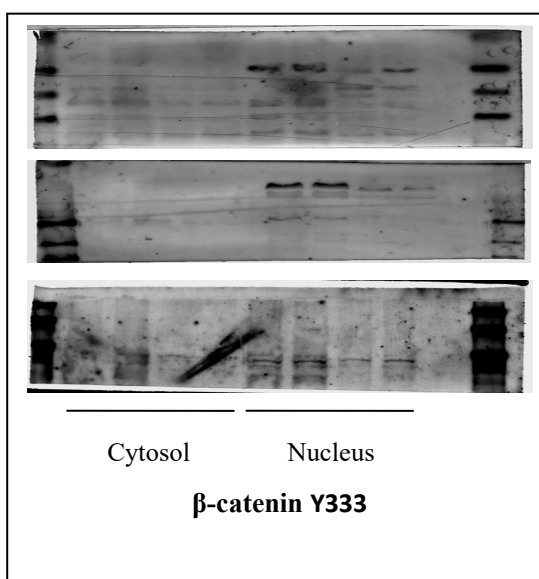

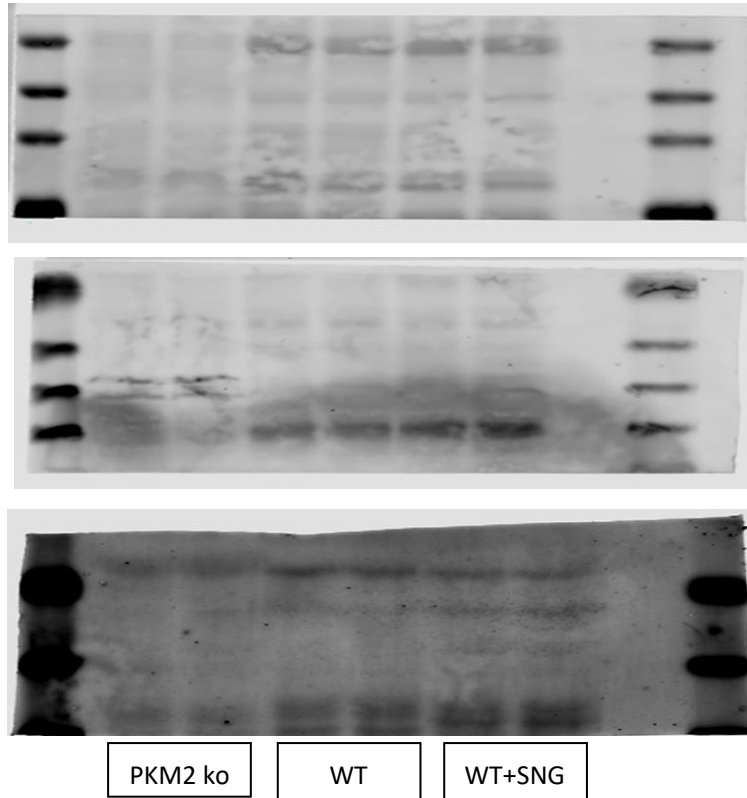

**Animal PKM2**

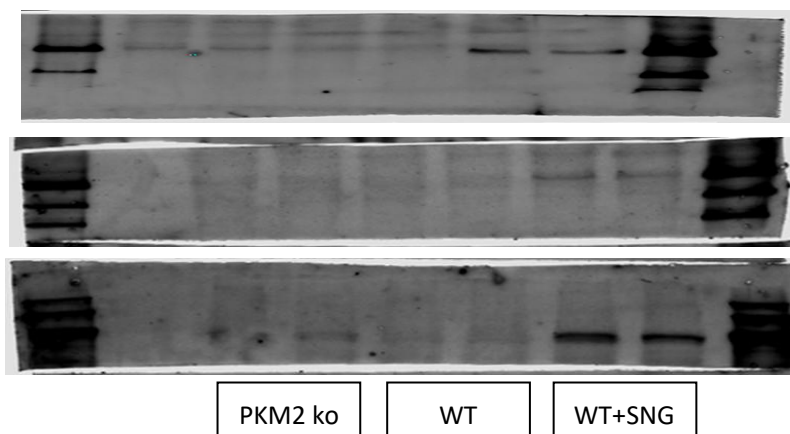

**Animal pMLKL**

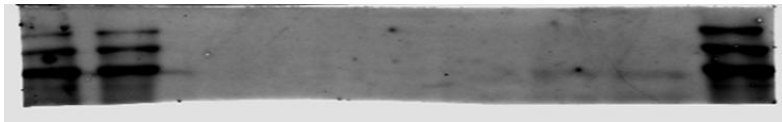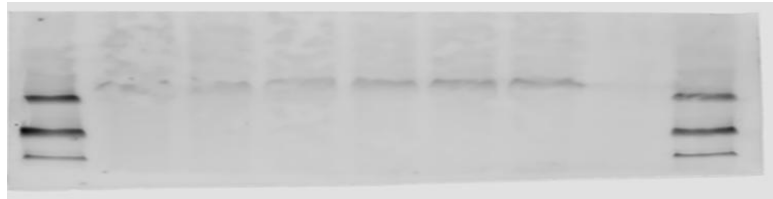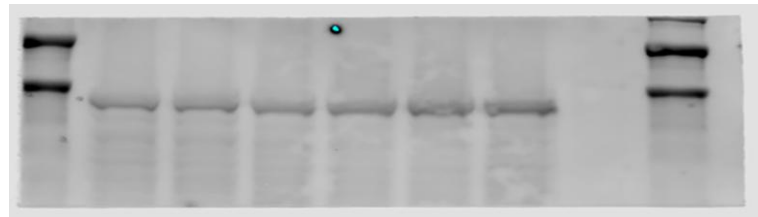

PKM2 ko

WT

WT+SNG

**Animal pRIP3**

Supplement: Supplementary file 1 [file cancers-16-02533-s001.zip › cancers-3051929-File S1.pdf]
